# Supplementary material for: Effect of Neuroprotective Magnesium Sulfate Treatment on Brain Transcription Response to Hypoxia Ischemia in Neonate Mice
Source: Int J Mol Sci. 2021 Apr 20;22(8):4253. doi: 10.3390/ijms22084253 (PMC8074012; doi:10.3390/ijms22084253)
Supplement: Supplementary file 1 [file ijms-22-04253-s001.zip › ijms-1189883-non-published/ijms-1189883-supplementary.pdf]

1. Material and Methods

1.1 Animals

The body weight at day 5 was different among acute or repeatedly sex-matched treated pups ($p < 0.0001$, according to Student's *t* test). This effect may be due to daily handling in repeatedly treated pups. Although only sex differences were observed in acutely treated pups. At weaning (P30), in acutely treated animals, sex and treatment were significant causes of differences in two-way ANOVA ($p = 0.0092$ and $p = 0.0002$, respectively). Only $MgSO_4$ 600 mg/kg treated male pups had higher body weight than controls. No significant differences were noted between females. Repeatedly treated pups (from P5 to P9) had significantly lower body weight than acutely treated animals and sex differences were significant using 2-way ANOVA ($p = 0.0385$ and $p < 0.0001$, respectively). Post-hoc analysis revealed decreased body weight in males (- 9.34%, $p < 0.001$) and in females (- 6.89 %, $p < 0.05$) treated by $MgSO_4$ relative to PBS repeatedly injected controls, as well as the difference between PBS treated males and females was also significant (- 4.56%, $p < 0.05$). In $MgSO_4$ treated groups, no sex differences were detected. This data indicate that repeated injections of PBS had low effect on neonate growth from P5 to P30, but that $MgSO_4$ 600 mg/kg impaired this growth.

Activity in all tests differed in males and females. Thus, analyses were done in separated sex. The different tests were performed in 12 – 44 animals per group (Table S3).

1.2 Transcriptome studies

The lists of genes with expression affected by $MgSO_4$ and/or HI were submitted to DAVID® software (v 6.8) extraction of enriched GO terms (seGOterms), Kegg Pathways (seKPaths) and UP_Keywords (seKws) at $N \geq 10$ and False Discovery Rate (FDR) < 10% thresholds, and fulfilling one of the two following criteria; i) high significance indicated by the corrected Bonfferroni test p-value $< 1 \times 10^{-4}$, or ii) EASE p value $\leq 1 \times 10^{-4}$ and Fold Enrichment (FE) > 2. Redundancy was limited using Revigo® filtering on dispensability > 0.7. A separate analysis was performed unsing Ingenuity pathway analysis (IPA®).

Detailled lists of genes, seKws, seGO-terms, seKPaths, IPA ® pathways (seIPaths) and IPA® putative Upstream regulators (pUR) are provided in separate Excel Tables; effects of $MgSO_4$ single injection in Table S1.xlsx and interaction of $MgSO_4$ pre-treatment with transcription response to HI in Table S2.xlsx.

1.3 Behavior studies

The effects of $MgSO_4$ neonatal treatments (600 mg/kg single injection at P5) in grownups were assessed versus neonate PBS injection effects at adulthood (60-90 days) and compared to pharmacological treatments with known agents active at the NMDA type glutamate receptor antagonists (dizocilpine (MK-801), 1 mg/kg) or ketamine (40 mg/kg) administered once at P5, in order to assess a putative interaction of exogenous Mg^{2+} ion at NMDA receptor. In addition two groups exposed to repeated $MgSO_4$ administration (600 mg/kg dose for 5 days (P5-P9), or PBS were constituted. All groups were explored in a series of spontaneous to highly integrated behavior tests: Spontaneous activity in home cages with running-wheel, locomotor activity and exploration in an open field device, pharmacological MK-801 induced hyperactivity and social interaction. Sex effects were evaluated in all tests.

1.3.1 Spontaneous activity in the wheel

Spontaneous activity in running wheel was recorded for 3 nycthemeres (start at 14h00) under standard housing. Distance ran in wheel was sampled every 10 min, number of courses and direction were recorded on the 72h, as previously reported [1].

1.3.2 MK-801-induced locomotor activity in adults

Neonatal blockade of NMDA transmission impairs brain development and result in long term behavior deficits. The test at adulthood of MK-801 single injection-induced hyper-locomotion was performed to

assess putative deleterious interference of neonatal $MgSO_4$ treatment with NMDA transmission [2]. Locomotor activity was measured in the open-field device (60 x 60 cm) for 180 min at P60-90, using a Versamax 4.2 apparatus (Accuscan Instruments Inc. Ohio, (USA) device, based on infrared beam crossing). Two criteria were followed; total distance ran and exploration of center part of the open field was used as an index of des-inhibition for initial 30 min. After 30 min the animals received a 375 µg/kg i.p injection of MK-801 to induce locomotor activity. This activity was followed for 150 min. The parameters recorded were total distance, vertical activity and stereotypies.

Data were analyzed using two-way ANOVA (sex/treatment) and Bonferroni post-test, on the treatment effects in each sex in the 8 acutely neonatal treatment groups (sex + treatment), and separately in the 4 repeatedly-treated groups of neonates, using GraphPad Prism6® software (La Jolla, CA, USA).

1.3.3 Social interaction

Social interaction test device consist in a black box separated in two compartments by a grid as previously described [3]. The large compartment (42 x 12 cm) receives the test animal for a 3-min habituation period. Then an unknown congener (non-treated, same sex) is inserted opposite to the grid in the small compartment (12 x 12 cm) for a 6-min interaction period. A video-tracking (ANYMaze®) allowed to analyze test animal behavior over virtual subzones named contact, proximal, intermediate and distal in reference to the grid. In each group, the parameters recorded for statistical analyses were time spent, rearing and distances covered in the all field and inter-zone crossings, in both habituation and interaction periods. The ratio of interaction to habituation scores was considered the induction of effect due to the unknown congener. Time spent and entries were measured in virtual sub-comparments (contact to distal from the congener). More specifically, nose poke events, nose poke total and mean duration were analyzed during interaction period.

A total of 135 mouse neonates at 5 days received single injection of PBS, MK-801 (1 mg/kg), ketamine (40 mg /kg) or $MgSO_4$ 600 mg/kg. In addition, 69 pups were injected daily from 5 to 9 days included with PBS or $MgSO_4$ (600 mg/kg). An overall mortality of 2.13% was detected in pups at 3 weeks (weaning). A low mortality was observed in MK-801 treated males (10%), and in ketamine treated males (7.14%).

Sex differences were noted in most parameters under evaluation. Thus data were treated separately. The analyses were conducted in several steps for each behavioral trait recorded. Two groups of criteria were evaluated. Activity criteria (total distance browsed, interzone crossings, rearings and distribution of activity related to the grid estimated by the ratio of time spent in distal + intermediate to proximal zones). These criteria were examined in both habituation and interaction periods. Changes in distribution of these activities in the two periods were investigated and referred to as "induction". A second group of criteria was investigated in the interaction period only and concerned specific interaction parameters (nose poke numbers, time and interaction time). The analyses were done in the 4 groups of mice (males or females) having had acute treatments were analyzed using one way ANOVA followed by Bonferroni post-test. A two-way ANOVA was performed to extract congener induced changes in activity parameters on period (habituation/interaction) and treatment, followed by Bonferroni post-test. The putative effects of neonatal treatments on the spatial distribution of activity within the maze was evaluated using the Chi^2 square test of inter-zone crossings since test requires whole numbers (no mean, no decimal). Activity distribution in specific group was compared to the activity distribution of all animals, respective of sex and treatment type (acute or repeated).

1.5 Interference of MgSO4 single injection with spontaneous development

In a previous study we reported spontaneous developmental expression variation in the whole genome from P2 to P15 (GEO accession GSE144456. Go to https://www.ncbi.nlm.nih.gov/geo/query/acc.cgi?acc=GSE144456 [3].

Here we extracted genes affected by $MgS0_4$ and developmentally regulated on the P2 to P10 period. Owing that both senses variations occurred either in $MgSO_4$ effects and ontogenetic variations, and that ontogeny was recorded over 2 periods (P2 to P5 and P5 to P10) we extracted $2^3 = 8$ interaction subgroups. We regrouped $MgSO_4$ induced variation in the sense of ontogeny; i) amplification of previous ontogenetic effect or anticipation of subsequent fate; group called ORTHO, or ii) opposite to the sense of ontogeny, i.e. reversion of previous ontogenetic effect or "prevention of subsequent fate; group called CONTRA.

Table S3. Number of animals per experimental group

	Acute PBS	Acute MK-801	Acute Ketamine	Acute MgSO4	Repeated PBS	Repeated MgSO4
**Spontaneous locomotion in running wheel**						
Males	44	28	29	25	13	13
Femelles	39	24	18	26	14	16
**MK-801 Induction of locomotion**						
Males	13	18	13	13	14	13
Femelles	13	18	12	12	14	17
**Social behavior**						
Males	17	17	21	12	15	14
Femelles	17	18	21	12	19	21
**Transcriptome study**						
	Ctrl[§] (Mg)	$MgSO_4$	Ctrl (HI)	HI	MgHI	
Males	45*	45	18[#]	18	18	
Femelles	45	45	18	18	18	
Sex matched	90	90	36	36	36	

[§] Mg and HI respective controls were performed separately, from respective liters. MgHI *vs* Ctrl one color analysis was done using pooled Ctrls from the two groups.
* groups of 45 were made of 3 individual hemispheres x 5 time points x 3 replicates.
[#] groups of 18 were made of 3 individual hemispheres x 2 time points x 3 replicates.

2. Results

2.1 MgSO4 proper effects

2.1.1 Transcription studies (List A)

David® (v6.8) analysis of List A identified 21 seKw, showing Bonferroni p value $< 1 \times 10^{-4}$, FDR < 10% and at least 2 fold enrichment. This short series includes 1057 different genes (75% of total List A) exhibiting 4.8 fold more repressions than in the total list A (2.18 fold more repressions) ($p = 8.34 \times 10^{-5}$ according to Fisher exact test (OR = $2.27_{(1.452 – 3.486)}$, indicating that $MgSO_4$ had mainly convergent repression effects (Table S1B).

Amongst the 1411 genes in List A, 517 genes (36.6 %) were associated to enriched Keywords, GO terms or pathways selected under high stringency. The predominance of repressions observed in List A was even more striking among genes associated to enriched terms (Odd ratio (O.R.) > 2 in all terms), indicating coordinated effects of $MgSO_4$ treatment towards repressions. Reciprocally $MgSO_4$ effects on genes non associated to these terms included less proportion (O.R. = 0.65) of repressions (Figure S1B).

DAVID® extraction of seGO-terms from the 1411 genes affected by $MgSO_4$ single injection (List A) followed by ReVIGO selection isolated 43 seGOterms, distributed in 16 Biological Process, 13 Cell compartments and 14 Molecular Functions terms. A total of 716 genes among the 1411 list (50.7%) contributed to seGOterms enrichment, of which 136 genes were induced, 573 genes (80%) were repressed and 7 exhibited putative splicing (Table S1B).

One remark that seGOterms focus towards several main features (some terms could be associated with one or more features).

- Eleven seGOterms were related to nervous system development and synapses (GO:0007399, GO:0048167, GO:0050885, GO:0031338, GO:0045202, GO: 0030054, GO:0043005, GO:0014069, GO:0042995, GO:0005912, GO:0098641)
- Seven seGOterms were related to cytoskeleton (GO:0030036, GO:0006897, GO:0005856, GO:0005874, GO:0030027, GO:0055038, GO:0008017)

- Six seGOterms were related to kinases and phosphorylation processes (G0: 0006468, GO;0046777, GO:0016310, GO:0018105, GO:0004672,GO:0019901)
- Four seGOterms were related to regulation of transcription (GO:0000122, GO:0003714, GO:0003682, GO:0000977)
- Four seGOterms were related to signaling (GO:0035023, GO:0005089, GO:0005096, GO:000 5516)
- The remaining was associated to general cell processes (memory, apoptosis or cell migration) or generic biochemical processes (Regulation of catabolism, SH3 binding), localisation (perinuclear region of cytoplasm and golgi apparatus (GO:0000139, GO:0005794).

DAVID® Analysis allowed to extract seKPaths at a high significant level, according to the restrictive Bonferroni test (Table S1C). Of note the highest significance was related to axon guidance, reminding the previously described seGOterms associated to nervous system development. Netrin signaling (also associated to axonal guidance), reelin neuronal migration cue signaling system completed this series of nervous system development related pathways (Table S1B,C). To a lower significance, although detected with p value < 0.05 according to EASE, we could observed several pathways related to neurotransmission and signaling (Table S1C).

Ingenuity Pathway analysis on $MgSO_4$ proper effects at the different time points revealed early onset of many coordinated effects towards seIPA-paths. The "synaptogenesis signaling pathway" was the highest significant seIPA-path ($p = 4.78 \times 10^{-9}$).Forthy seIPA-paths had z-score below -2 at least at one time point and only one a z-score above 2 (PTEN signaling), indicating coordinated slowering effects of $MgSO_4$ on transcription in many cell functions. (Table S1D).

IPA upstream regulator research showed Mnk1 inhibition was a very significantly in the entire 24h period, culminating 6h after $MgSO_4$ (Table S1E) and affecting up to 30 genes (minimum p-value = 2.15×10^{-15} and minimum z-score = -5.2). Frm-1 activation from 90 min to 24h after $MgSO_4$ injection affected for up to 27 genes (minimum p-value = 3.34×10^{-8} and z-score = 2.84). This positive z-score indicated Fmr-1 activation, but essentially resulted in downstream gene repressions, especially those included in synapto-genesis, reelin and glutamate signaling pathways. Interestingly, a very small proportion of downstream effects of Mnk1 and Frm1 were common (n = 4), indicating that putative $MgSO_4$ inhibition of these functions resulted from additive effects through at least 2 distinct cooperating targets (Mnk1 and Frm-1).

2.1.2 $MgSO_4$ effects on genes spontaneously regulated during P2-P10 development

$MgSO_4$ treatment affected few genes spontaneously regulated in the P2-P5 period, former to injection (n = 45) although, it affected expression of many genes (n = 254) committed to be regulated in the subsequent normal development (Figure S2, Table S1F).

The vast majority of $MgSO_4$ transcription effects gathered in the ORTHO group (n = 290). Near 60 % of genes in the ORTHO group belong to a wave of transient inductions or repressions occurs in brain development from P5 onwards [3]. Of note, inductions (n = 125) predominated repressions (n = 48), although the majority of overall $MgSO_4$ effects was repression. Only a few genes exhibited $MgSO_4$ induced regulation constituting the CONTRA group (n = 33). On the whole, it appears that MgSO4 prolonged and mainly anticipated normal developmental changes, although affecting a modest proportion of developmentally regulated genes around P5 (323/2319 = 13.9%).

HI in P5 mouse brain had little effect on the expression of genes spontaneously evoluting along development in days before or after P5, mostly opposite to what is committed to happen in the 5 to 10 days period (Fig S2B), confirming previous data [3]. The interference of MgHI (List B) with ontogenically regulated genes appeared as an amplification of HI effects, including the majority of genes affected by HI alone (Fig S2C). These effects thus appeared opposite to ORTHO effects induced by $MgSO_4$ single injection (Table S2F).

2.1.3 Behavior studies

2.1.3.1 Spontaneous activity in wheel

Two-way ANOVA analyses of acute or repeated treatments effects (sex/treatment) revealed sex effect; females being more active than males.

$MgSO_4$ neonatal treatment (600mg/kg) either after single or repeated injections did not affect spontaneous running activity in the wheel. (Figure S3A,B). A tendency was noted in males ($p = 0.20$). Of note, repeated handling in neonates resulted in reduced activity in adults whatever the sex and the treatment. No differences were observed on the number of running cycles and direction (not shown).

As previously described, ketamine increased the activity in females [1]. No MK-801 effect was observed.

Table S4: Distance in running wheel

**Spontaneous locomotion in running wheel**		Acute treatments		Repeated treatments	
72h Distance in the wheel					
2-way ANOVA	Sex effect	p < 0.0001	***	p = 0.0002	***
	Treatment effect	p < 0.0001	***	p = 0.5879	
	Interaction	p = 0.0726		p = 0.3422	
Bonferroni post-test	Males	ns#		ns	
	Females	Ketamine p < 0.0001	***	ns	

ns indicate no treatment effects in sex separated groups,

***; $p < 0.001$.

2.1.3.2 Actimetry-Open field

Total distance and time spent in the center of the open field were registered for the 30 min habituation time. Significant sex differences were observed in both criteria and treatment type (acute or repeated). No treatment effect was observed. Only interaction between sex and treatment appeared significant in the center part exploration time in repeated treatment groups (Figure S3C-F and Table S5).

Table S5: Open Field exploration

Open Field		Acute treatments		Repeated treatments	
30 min exploration: Total distance					
2-way ANOVA	Sex effect	p = 0.0012	**	p = 0.0079	**
	Treatment effect	p = 0.7121		p = 0.1743	
	Interaction	p = 0.9354		p = 0.6264	
Bonferroni post-test	Males	ns#		ns	
	Females	ns		ns	
30 min exploration: Center time					
2-way ANOVA	Sex effect	p < 0.0001	***	p = 0.0003	***
	Treatment effect	p = 0.2970		p = 0.1970	
	Interaction	p = 0.8920		p = 0.0287	*
Bonferronni Post-test	Males	ns		ns	
	Females	ns		ns	

ns indicate no tretatment effects in sex separated groups,

*; $p < 0.05$, **; $p < 0.01$, ***; $p < 0.001$.

2.1.3.3 MK-801 induced hyperactivity

The response to acute MK-801 was assessed using three parameters recorded for 150 min; total distance ran recorded for 150 min, vertical exploration inhibition recorded for every 5 min period in which it was below 15 min pre-injection level, and stereotypies. Acute and repeated neonatal effects were evaluated separately versus respective PBS administration schedule. Two-way ANOVA evaluating sex and treatment effects and putative interaction were performed at first. Since quantitative values of activity mostly differed in males and females, subsequent analyses were done in separated sex using one way ANOVA and Tuckey post-test.

The three parameter evaluated distinct aspects of MK-801 induction effects as revealed by two-way ANOVA in acutely neonatal treated groups. Two-way ANOVA revealed sex differences in total distance ran induction, treatment effects on vertical exploration (repression) and interaction between sex and treatment on

Table S6: Acute MK-801 induction effects.

Open Field		Acute treatments		Repeated treatments	
150 min period: Total distance					
2-way ANOVA	Sex effect	p = 0.0023	**	p = 0.7172	
	Treatment effect	p = 0.6088		p = 0.4150	
	Interaction	p = 0.3171		p = 0.7890	
Bonferroni post-test	Males	ns[#]		ns	
	Females	ns		ns	
150 min period: Vertical exploration					
2-way ANOVA	Sex effect	p = 0.4931		p = 0.3464	
	Treatment effect	p = 0.0080	**	p = 0.3018	
	Interaction	p = 0.7200		p = 0.1231	
Bonferroni post-test	Males	MK-801[$] p = 0.0116	*	ns	
	Females	ns		ns	
150 min period: Stereotypies					
2-way ANOVA	Sex effect	p = 0.1752		p = 0.4807	
	Treatment effect	p = 0.5753		p = 0.1334	
	Interaction	p = 0.0053	**	p = 0.9758	
Bonferroni post-test	Males	MK-801 p = 0.0018	**	ns	
	Females			ns	

[#] ns indicate no treatment effects in sex separated groups,
[$] Effect of neonatal MK-801 *vs* neonatal PBS,
*; p < 0.05, **; p < 0.01.

induction of stereotypies (Table S6). Post-test analyses revealed a significant effect of neonatal MK-801 treatment in males on the inhibition of vertical exploration and induction of stereotypies acute induction by subcutaneous MK-801 in adults (Figure S4). In females a tendency towards reduction of stereotypies was observed although it did not reach significance (p = 0. 0921). This variation in the opposite sense to the observation in males was responsible for the interaction observed on stereotypies (Table S6). Of note repeated neonatal $MgSO_4$ treatments had no effects in these paradigms.

2.1.3.4 Social interaction

No effect of acute neonatal treatments on exploration features in the 2-compartment device could be detected during habituation periods, although sex differences were patent in many criteria. During interaction period sex differences appeared highly significant in both acute and repeated treatment studies (Table S7). Considering the number of interzone crossings in habituation and interaction periods, it appeared than the introduction of the unknown congener provoked an increase in the entries in contact zone in most groups (main text Figure 4). The analysis by Chi^2 of entry distributions revealed that acute treatments altered the exploration pattern; specifically ketamine had highly significant effect (Table S7). MK-801 showed a nearly significant effect. The effect of neonatal ketamine appeared as a strong reduction of congener induced increase in contact-zone entries. Of note contact-zone time was not modified (not shown), indicating that ketamine modified, although did not abolished social interaction.

Table S7: Social interaction.

Open Field		Acute treatments		Repeated treatments	
3 min habituation: Time in contact zone					
2-way ANOVA	Sex effect	p = 0.0240	*	p = 0.0653	
	Treatment effect	p = 0.0568		p = 0.7000	
	Interaction	p = 0.6598		p = 0.4489	
Bonferroni post-test	Males	ns[#]		ns	
	Females	ns		ns	

6 min interaction: Time in contact zone					
2-way ANOVA	Sex effect	p = 0.0008	***	p = 0.0015	**
	Treatment effect	p = 0.0859		p = 0.8879	
	Interaction	p = 0.7763		p = 0.4902	
Bonferroni post-test	Males	ns		ns	
	Females	ns		ns	
3 min habituation: Interzone crossings induced by congener					
Chi^2		(Chi^2, Df, p)		(Chi^2, Df, p)	
	Males	14.26, 9, 0.1135		4.059, 3, 0.2552	
	Females	15.43, 9, 0.0797		4.425, 3, 0.2191	
6 min interaction: Interzone crossings induced by congener					
Chi^2		(Chi^2, Df, p)		(Chi^2, Df, p)	
	Males	11.89, 9, 0.2196		2.333, 3, 0.5063	
	Females	31.15, 9 0.0003	***	1.723, 3, 0.6319	
Fisher Exact test	In Females				
	MK-801 *vs* PBS	p = 0.0856			
	Ketamine *vs* PBS	p = 0.0116	*		
	$MgSO_4$ *vs* PBS	p = 0.6712			

[#] ns indicate no treatment effects in sex separated groups,

*; p < 0.05, **; p < 0.01, ***; p < 0.001.

2.2 $MgSO_4$ effects on HI (List B)

The modulation by $MgSO_4$ of HI transcription effects cannot be described by the sole analysis of list ②, as this list also contained $MgSO_4$ proper effects and HI-effects only detected in Ctrl. The modulation by $MgSO_4$ of effects observed in HI non pre-treated animals only represented a minor proportion of MgHI effects (Figure S5). The $MgSO_4$/HI interfering effect was therefore given by the analysis of List B that resulted from the concatenation of observations in several experiments. List B was constructed to identify expected aspects of $MgSO_4$ effects; reversion of HI effects (List B2), amplification of HI effects (List B3). One could observe the occurrence of a major series of so-called revealed genes only observed after $MgSO_4$ pretreatment in HI-exposed brain (List B1) and representing more than ¾ of modulations (main text Figure 6A). Reversions of HI effects represented near ¼ of the total and amplifications by Magnesium of HI effects and were 1.2% of total List B (main text Figure 6C). $MgSO_4$ effects in HI brains (List B) provoked near twice as much inductions than in Ctrl mice representing 44.4% of regulations; a significantly higher proportion than in Ctrl brains (31.5%) (p = 1.68E-12 (OR = $0.597_{(0.516\text{-}0.691)}$ according to Fisher exact test) (Table S2A).

2.2.1. Prevention by $MgSO_4$ of HI effects

List B_2 regrouping prevention of HI effects by $MgSO_4$ pretreatment constituted near a quarter of list B (main text Figure 6C). A total of 137 genes with expression reversed by $MgSO_4$ pretreatment only represented 23.8 % of the effects of HI in list ③. Of them 118 were repression by $MgSO_4$ of HI inductions in Ctrl, and thus only 19 inductions by $MgSO_4$ of genes repressed after HI in Ctrl. Mg pretreatment therefore appears as partial prevention of inflammation/immunity and transcription regulation at RNA polymerase II processes due to HI (Table S8, Figure S6).

2.2.2 de novo HI-induced regulation after MgSO4 pretreatment

List B1 regrouped de novo occurrences of genes not regulated at a significant statistical level in $MgSO_4$ solely injected mice nor in HI exposed Ctrl mice. DAVID® analysis of de novo HI-induced regulation after MgSO4 pretreatment was based on extraction from List ⑦ obtained by one-color analysis and to MgHI effect *vs* Ctrl of genes not affected by HI in List ③ (main text Figure 2). Among the 729 genes affected by HI and the 1178 genes affected by HI in $MgSO_4$ pretreated mice, only 140 were common and a major part of them were induced in both conditions (n = 101). 34 genes were repressed in both lists and 5 genes exhibited significant inverse response in absence or pretreatment by $MgSO_4$ (representing a small proportion of all

Table S8 : Comparative enrichment in keywords, and GO-terms the effects of $MgSO_4$ treatment and/or HI in neonate mice, at the Bonferroni p values < 1x 10^{-4} in at least one list.

**Keywords**	**NB**	**p value**	**FE**	**NB**	**p value**	**FE**	**NB**	**p value**	**FE**	**NB**	**p value**	**FE**	**NB**	**p value**	**FE**
Term	**List A** : Proper effects of $MgSO_4$			**List B** : MgHI vs HI effects			**List ③** : Effects of HI			**List ⑦** : MghI vs Ctrl One-color analysis			**List C** : HI effects unaffected by Mg		
Phosphoprotein	732	2.15E-56	1.62	808	5.62-36	1.44	319	4.60E-10	1.38				108	7.71E-04	1.45
Ubl conjugation	152	3.00E-8	1.70	161	5.29E-4#	1.45	74	1.45E-2#	1.62				31	*1.51E-04*	2.10
Transcription regulation	161	3.84E-5	1.51	187	4.06E-4#	1.41	92	2.72E-4#	1.69				39	*4.73E-06*	2.21
Alternative splicing	435	4.95E-21	1.53	468	1.01E-9	1.33	213	1.93E-7	1.47						
Apoptosis				71	2.67E-5	1.97	35	2.24E-3#	2.36	71	2.67E-05	1.97	14	*1.07E-03*	2.92
Differentiation	76	7.18E-6	1.98				42	2.43E-3#	2.15						
Methylation	128	1.66E-15	2.25										17	*6.24E-04*	2.69
Neurogenesis	48	3.56E-10	3.28							36	*1.47E-04*	1.98			
Cell junction	102	2.90E-16	2.60												
Synapse	70	5.08E-16	3.31												
Golgi apparatus	99	5.52E-11	2.22												
Cell projection	82	5.09E-7	2.04												
Transferase	153	1.08E-5	1.56												
Zinc-finger	146	1.45E-5	1.57												
Kinase	79	3.29E-5	1.88												
Cytoskeleton	113	1.15E-6	1.78												
Activator				88	2.52E-6	1.91	44	1.73E-4#	2.33	88	2.52E-06	1.91	16	*1.25E-03*	2.62
Transcription				191	6.93E-4#	1.39	94	3.34E-4#	1.67				40	9.49E-4	2.20
Mitochondrion				176	1.88E-22	2.26				176	1.88E-22	2.26			
Mitochondrion inner membrane				45	3.80E-5	2.40				45	3.80E-05	2.40			
Ribonucleoprotein				54	2.42E-6	2.38				54	2.42E-06	2.38			
Transit peptide				77	1.26E-6	2.05				77	1.26E-06	2.05			
Nucleus				470	4.63E-14	1.41	186	1.56E-3#	1.35						
Cytoplasm				483	5.33E-20	1.49	177	1.00E-2#	1.33						
Acetylation				417	2.01E-34	1.81				417	2.01E-34	1.81			
**GO terms**	**NB**	**p value**	**FE**	**NB**	**p value**	**FE**	**NB**	**p value**	**FE**	**NB**	**p value**	**FE**	**NB**	**p value**	**FE**
Term	**List A** : Proper effects of $MgSO_4$			**List B** : MgHI vs HI effects			**List ③** : Effects of HI			**List ⑦** : MghI vs Ctrl One-color analysis			**List C** : HI effects unaffected by Mg		
positive regulation of transcription from RNA polymerase II promoter							71	6,09E-09	2.09				29	*4.60E-06*	2.64
transcription regulatory region DNA binding							26	3,78E-07	3.29						
circadian rhythm							17	7,53E-07	4.61				8	*1.92E-04*	6.70
transcriptional activator activity, RNA polymerase II core promoter proximal region sequence-specific binding							27	1,76E-06	2.95						
nuclear chromatin							24	1,91E-06	3.20						
transcription factor binding							31	2,06E-06	2.67						
cell chemotaxis							13	1,22E-05	4.88						
cellular response to IL-1							13	1,59E-05	4.76						
transcription from RNA polymerase II promoter							17	1,93E-05	3.61				17	*2.23E-05*	3.25

angiogenesis							23	2,44E-05	2.82						
response to lipopolysaccharide							20	4,58E-05	2.98						
positive regulation of inflammatory response							11	4,91E-05	5.12						
double-stranded DNA binding							16	5,85E-05	3.47						
response to cytokine							12	9,13E-05	4.34				10	*3.47E-05*	8.93
transcription, DNA-templated							95	9,49E-05	1.48				8	*3.07E-05*	6.55
Axon guidance	91	2,69E-16	2,80												
Thyroid hormone signaling pathway	75	1,01E-12	2,77												
Neurotrophin signaling pathway	49	8.14E-10	3.18												
cAMP signaling pathway	64	2.94E-08	2.56												
mitochondrion				776	2,61E-21	1,29				200	1.13E-21	2.08			
cytoplasm				703	1,10E-16	1,28	267	3.09E-05	1.23	491	3.43E-12	1.33			
nucleus				345	1,75E-13	1,42	245	4.32E-05	1.25	448	8.64E-11	1.33	93	*4.28E-05*	1.43
extracellular exosome				248	1,36E-09	1,53				222	2.50E-07	1.49			
cytosol				64	2,31E-09	2,20				185	1.81E-14	1.86	43	*9.07E-07*	2.24
intracellular ribonucleoprotein complex				746	2,48E-06	1,17									
membrane				43	1,02E-05	2,52									
ribosome				282	2,61E-21	1,80				39	2.17E-09	3.72			
protein binding				477	1,40E-06	1,27	203	1.24E-09	1.46	309	3.12E-06	1.34	81	1.85E-05	1.75
poly(A) RNA binding				161	5,94E-06	1,57				110	7.29E-06	1.76			
Translation										50	*2.25E-07*	2.23			
Structural constituent of ribosome										39	9.86E-05	2.62			
**KEGG pathways**	**NB**	**p value**	**FE**	**NB**	**p value**	**FE**	**NB**	**p value**	**FE**	**NB**	**p value**	**FE**	**NB**	**p value**	**FE**
Term	**List A** : Proper effects of $MgSO_4$			**List B** : MgHI vs HI effects			**List ③** : Effects of HI			**List ⑦** : MghI vs Ctrl One-color analysis			**List C** : HI effects unaffected by Mg		
Axon guidance	28	1,66E-05	3,27												
Thyroid hormone signaling pathway	25	8,09E-05	3,30												
mmu03010:Ribosome				30	*5,46E-05*	2,22				31	3,40E-07	3.57			
mmu05012:Parkinson's disease				30	*9,13E-05*	2,16									

* ; Bonferroni corrected p Value, # ; *Fisher p value* < 1E-4

reversions (n = 188). B_1 list in fact contributed to the major part of List B. Of note seKw, seGO-terms, and seKPaths were related to ribosome and to mitochondria (Table S2B-D, S8).

It can be postulated from observations of such large amounts of revealed regulations and from their specific spectrum, that the presence of Mg^{2+} in excess within the nervous tissue at the time when HI happened provoked an original transcription response to HI, different of what would happen in untreated animals.

2.2.3 Amplification effects

List B_3 showed the enhancement of only 18 induction and 5 repression (Table S2A). Three genes highly induced by HI showed also large enhancement *Ehbp1*, involved in cytoskeleton organization, *Peg10* also involved in cell shape and *xaf1* a regulator of apoptosis. The highest amplification of repression affected *Rprd1b*, an inducer of transcription by RNA polymerase II. List B3 was too small for enrichment analyses, but it appears that very few genes in B3 appeared in seKw, seGO-terms or seKPaths indicating that these sporadic effects likely result from chance.

2.3.4 Gene Ontology

Global analysis of list B using DAVID® extraction of enriched GO terms from the 1958 genes affected by $MgSO_4$ single injection (List B) followed by ReVIGO selection isolated 18 seGO-terms, distributed in 1 Biological Process, 13 Cell compartments and 4 Molecular Functions terms. A total of 1668 genes among the 1958 list (85.2%) contributed to seGOterms enrichments, of which 723 genes were induced, 914 genes were repressed and 31 exhibited putative splicing (Table S2C). Of note, although List B was larger than list A and List C, the number of enriched features was lower than in A or C. The enrichment of genes encoding mitochondrial and ribosomal proteins appeared as very specific to MgHI effects. These regulations were largely negative.

seKw extraction in List ⑦ largely converged with List B seKw, and poorly in the direction of HI effects per se (obtained from List ③ i.e. apoptosis, chemotaxis or angiogenesis, (Table S2B) or previously described [3]. It is remarkable that the major inflammation, immunity or transcription through RNA-polymerase II were absent from seKw from MgHI vs Ctrl inductions, indicating at least a global anti-inflammatory effect of $MgSO_4$ treatment (Table S8).

seGO-terms enrichment in List ⑦ recapitulating MgHI effects compared to basal level in Ctrl P5 mice also pointed on mitochondrion and ribosomes (Table S2C). Also, seGO-terms from List B_1 of $MgSO_4$ revealed HI effects included CC terms related to mitochondrion and ribosome, and the BP term GO:0006412~translation and the MF terms GO:0003735~structural constituent of ribosome and GO:0044822~poly(A) RNA binding. Altogether these observations indicate that protein translation should be affected by HI after $MgSO_4$ pretreatment whereas it was not in Ctrl mice.

More seIPAths were detected in MgHI (62) than in HI (41) mice, and 33 were observed in the 2 groups (Table S8). Two pathways had common enrichment at 3h only (IL-8 signaling, and RhoGDI signaling) and one at 12h only (Signaling by Rho family of GTPases). But, the transcription repression in MgHI group was observed early. Thirthy two (on 33) seIPaths in this group were only detected at the 3h time point. In addition among these 33 HI and MgHI common paths, 14 enrichments were anticipated in MgHI and 11 Pathways appeared only enriched at 3h (Table S2D).

Fourteen seIPA-paths were detected from list ②, all being repressed indicating a regulatory effect of $MgSO_4$ pretreatment towards HI induced transcription. Indeed, several of these pathways could also be observed out of $MgSO_4$ revealed HI transcription responses (List B_1) (Table S2D).

2.3 Tracking of magnesium targets

Many pUR showed opposite effects in Mg and HI groups, and did not remained in MgHI groups, suggesting an effect of $MgSO_4$ on these genes/proteins, it should be hazardous to assume that all of them were relevant targets of $MgSO_4$ modulating HI. Indeed, most downstream genes were common to several pUR (Table S2E, Figure S7).

Several genes involved in innate immune response; *MYD88, Tlr2 Lcn2* and transcription *Atf4,* appeared as pUR of $MgSO_4$ proper effects (List A) and HI-modulating effects (list ⑦). Similarly, two gene repressed by HI in Ctrl and in $MgSO_4$ appeared as apparent upstream regulators in different series; *Bcl3*; a regulator of NFκB and *Tet2*; a regulator of transcription and DNA demethylation. Among HI activated pUR that disappeared in MgHI groups, *Casr,* coding extracellular calcium receptor, may be a target through which $MgSO_4$ modulated HI effects since most genes downstream to *Casr* in HI were no more affected after MgHI (Figure S8). *Casr* transcription was not affected in any experiment.

z-score of several pUR appeared higher in MgHI than in HI suggesting amplification effect of Mg in the HI context (*Cdkn2a, Nr5a2, Dicer1, Ptpn1 and Gfi*) (Table S2E). In fact these pUR were based on different gene regulations in HI and in MgHI. None of these genes were in list B_3 of amplified transcription effects. The examination of pUR with lower z-scores, in MgHI conditions than in HI condition, could be supposed to be targets of Mg on HI effects prevention (Figure S8,S9). In this series, *Ifng* showed high proportions of specific putative targets (Table S2E).

The aim of IPA® extraction of pUR in the 9 lists was to identify the putative sites of $MgSO_4$ targets based on coherence between observations and known relationships (Details in Table S2E,F). 150 genes were identified as pUR in at least one of the nine lists of effects. Of them 83 exhibited complete reversion of z-score, indicating HI downstream effects prevented by MgSO4 pretreatment, thus designating them as Magnesium likely targets. Several pUR showed only a moderate effect on z-score, thus invalidating them as Magnesium effects. Upstream $MgSO_4$ effects could be transcriptional, although, few pUR showed a regulation of their own expression, in List A (8 were induced by $MgSO_4$ while 2 were repressed), in List B (8 were induced by $MgSO_4$ while 4 were repressed), and 11 in list ③(9 were induced by $MgSO_4$ while 2 were repressed) (Table S2E,F). Thus one should imagine that most $MgSO_4$ effects were non transcriptional, a feature in accordance with the rapid bio-availability of Mg^{2+}, and the early alteration of HI responses observed.

We have compared pUR in the nine lists according to common or opposite z-scores in the 3 main conditions; Mg, HI and MgHI (Table S2G, Figure S7). A lot of pUR were identified as putative inductors or repressors in HI and not in MgHI conditions, suggesting that $MgSO_4$ treatment prevented their HI-dependent activities (Fig S8-S9). Several pattern of $MgSO_4$ interference with HI can be distinguished based on pUR; i) disappearance in MgHI groups of pUR detected in HI, ii) remnance of pUR although with different characteristics (z-score, downstream genes panel) and iii) *de novo* emergence in MgHI groups of pUR not observed in HI groups. Not all pUR should be considered $MgSO_4$ actual regulation sites.

2.4 $MgSO_4$ insensitive effects of HI (List C)

DAVID® extraction of enriched GO terms was performed in List ③ confirming the evaluation of HI-transription effects previously observed [3], and in List C, to evaluate putative HI effects insensitive to $MgSO_4$ pretreatment. Gene induction predominated in HI, and contributed to enrichment in GO terms, Kegg Pathways and Keywords related to the transcription by RNA-polymerase-II, inflammation, angiogenesis. Reciprocally, repression of genes encoding cholesterol biosynthesis enzymes was noted for early steps (Idi1, Mvk and Mvd) and late steps (Msmo1 and Nsdhl), although this list did not reach significant thresholds according to DAVID® or IPA®. A minority of genes affected by HI (list ③; including 499 inductions and 224 repressions), appeared unaffected by pretreatment by $MgSO_4$ (List C; 166 inductions (33%) and 66 repressions (29%)). However, as far as gene ontology is valid for partial lists, extraction of seGOterms from list C indicated that the functions identified from List ③ remained (Table S8). Indeed, the analysis of $MgSO_4$ unaffected HI effects (List C) was based on a shorter list than the list of HI effects (list ③), but one could remark that most GO terms related to inflammation response were absent in C, in line with an anti-inflammatory effect of $MgSO_4$ treatment, although with the exception of response to cytokine and monocyte chemotaxis. pUR putatively activated after HI but did not appear in MgHI are presented in Figure S10.

2.5 Complementary MgHI vs Ctrl Hybridization control experiment

Crtl mRNA from experiment from experiment 1 and 3 were pooled and MgHI mRNA at 3h or 12h were used for co-hybridization in a supplement microarray. Probes exhibiting at least 2 fold change (whatever sense) or t test p-value $< 5 \times 10^{-2}$ associated to 1.5 minimum fold change were extracted as usual. 2159 genes were extracted (1220 inductions and 934 repressions) and submitted to seKw, seGO-terms and se-KPaths extraction using DAVID® using the following thresholds ($n \geq 10$, Bonferroni p value $< 5 \times 10^{-2}$ and FDR < 10%) or alternatively ($n \geq 10$, EASE p value $< 1 \times 10^{-2}$, Fold enrichment > 2 and FDR < 10%). Revigo filtering of GO trems allowed to extract non dispensable (dispensability < 70%) 19 BP-seGO-terms, 19 CC-seGO-terms and 19 MF-seGO-terms. In Addition, DAVID® allowed to identify 58 UP_keywords and 27 seKPaths. All did not appear relevant but one could observe:

- In UP_Keywords the terms: transcription, neurogenesis, mitochondrion, steroid biosynthesis, synaptosome, innate immunity, synapse and immunity,
- In BB-seGO-terms: “transcription, DNA templated”,”apoptotic process”, “nervous system development”,”positive regulation of inflammatory response”, “forebrain development” and “neuron migration”,

- In CC-seGO-terms: “neuron projection”, “mitochondrion”, “synapse”, “axon”, “post-synaptic density” and “dendritic spines”,
- In MF-seGO-terms; “transcriptional activator activity, RNA polymerase II core promoter proximal region” and “RNA polymerase II core promoter sequence-specific DNA binding”
- In seKPaths: “MAPK signaling pathway”, “neurotrophin signaling pathway” and “axon guidance”.

All these observations validated the data obtained in the core study by combination of two-color and one-color approaches (Data summarized in Table S2H).

3 References

1. Lecointre, M.; Vezier, C.; Benard, M.; Ramdani, Y.; Dupre, N.; Brasse-Lagnel, C.; Henry, V.J.; Roy, V.; Marret, S.; Gonzalez, B.J.; Jegou, S.and Leroux-Nicollet, I., Age-dependent alterations of the NMDA receptor developmental profile and adult behavior in postnatally ketamine-treated mice, Dev Neurobiol, 2015, 75:315-333.
2. Furuie, H.; Yamada, K.and Ichitani, Y., MK-801-induced and scopolamine-induced hyperactivity in rats neonatally treated chronically with MK-801, Behav Pharmacol, 2013, 24:678-683.
3. Dupré, N.; Arabo, A.; Orset, C.; Maucotel, J.; Detroussel, Y.; Hauchecorne, M.; Gonzalez, B.J.; Marret, S.; Vivien, D.and Leroux, P., Neonatal cerebral hypoxia-ischemia in mice triggers age-dependent vascular effects and disabilities in adults: impact of TPA, submitted, DOI.

4. Supplementary Figures

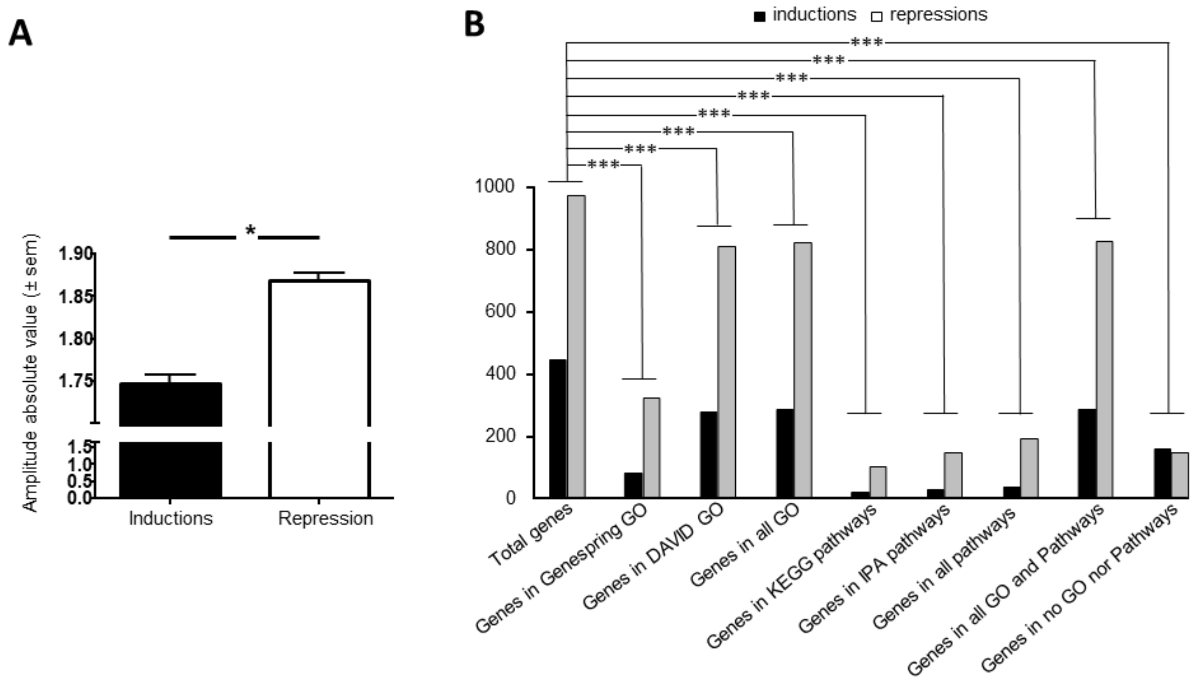

Figure S1. Comparison of amplitudes of Inductions and repressions after $MgSO_4$ treatment (**A**), and relative their relative distributions of inductions/repressions among genes included in enriched Keywords, GO-terms. Kegg and IPA pathways (**B**). * $p < 0.05$ and *** $p < 0.001$ according to Fisher exact test.

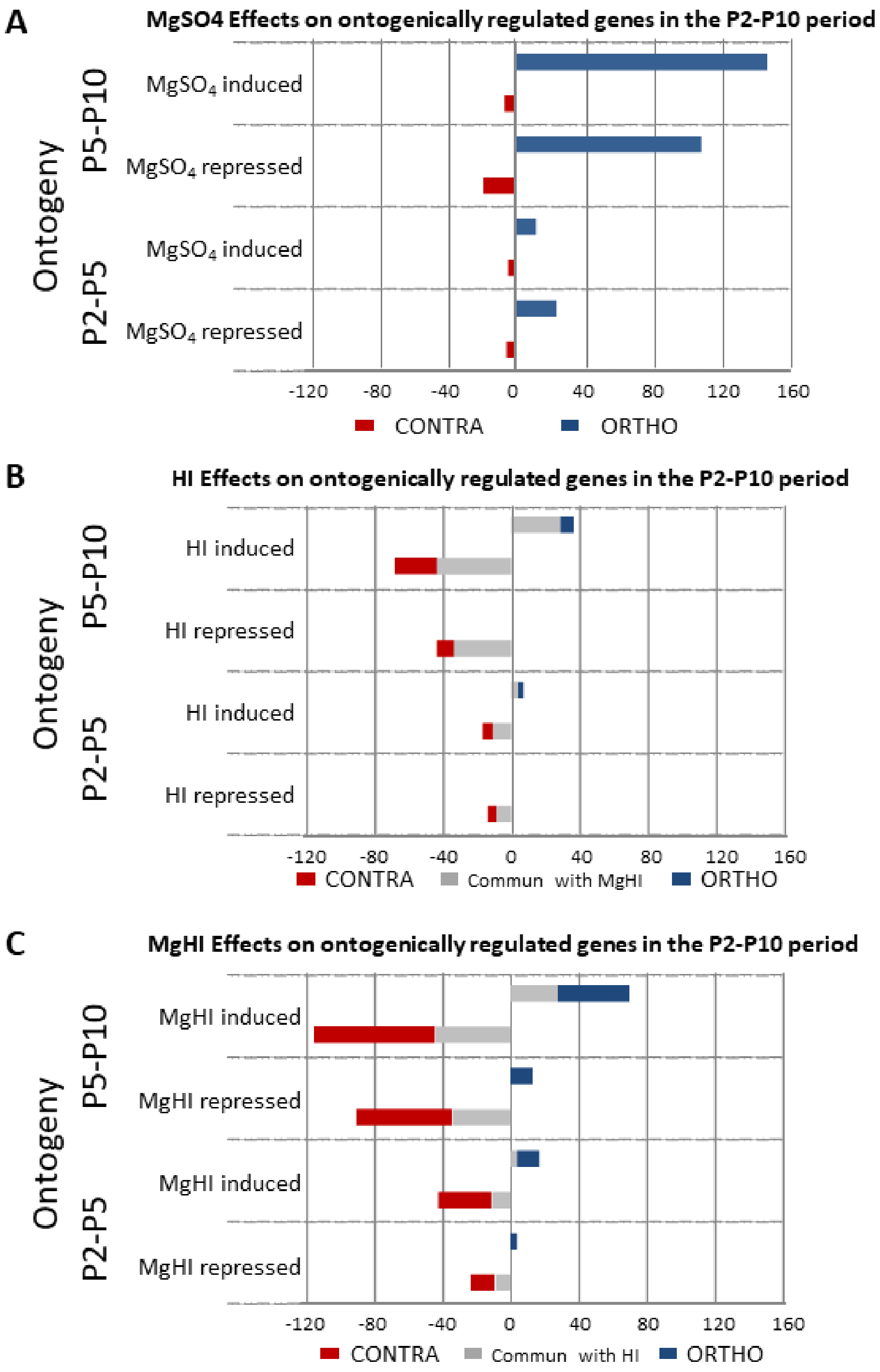

Figure S2. Comparison of gene numbers exhibiting transcription interaction with spontaneous ontogenic variation of $MgSO_4$ single injection (**A**), HI in Ctrl mice (**B**) and HI in $MgSO_4$ pretreated mice (**C**). The effects of different treatment evaluated on P2-P5 period (previous to treatment) or P5-P10 period (post-treatment) were classified according to variations in the sense of spontaneous development (named ORTHO) or antagonistic to development (names CONTRA, represented as negative values in left parts of the graphs). Parts of histograms in grey represent observations in common to HI in Ctrl (B) or MgSO4- pretreated (C) groups.

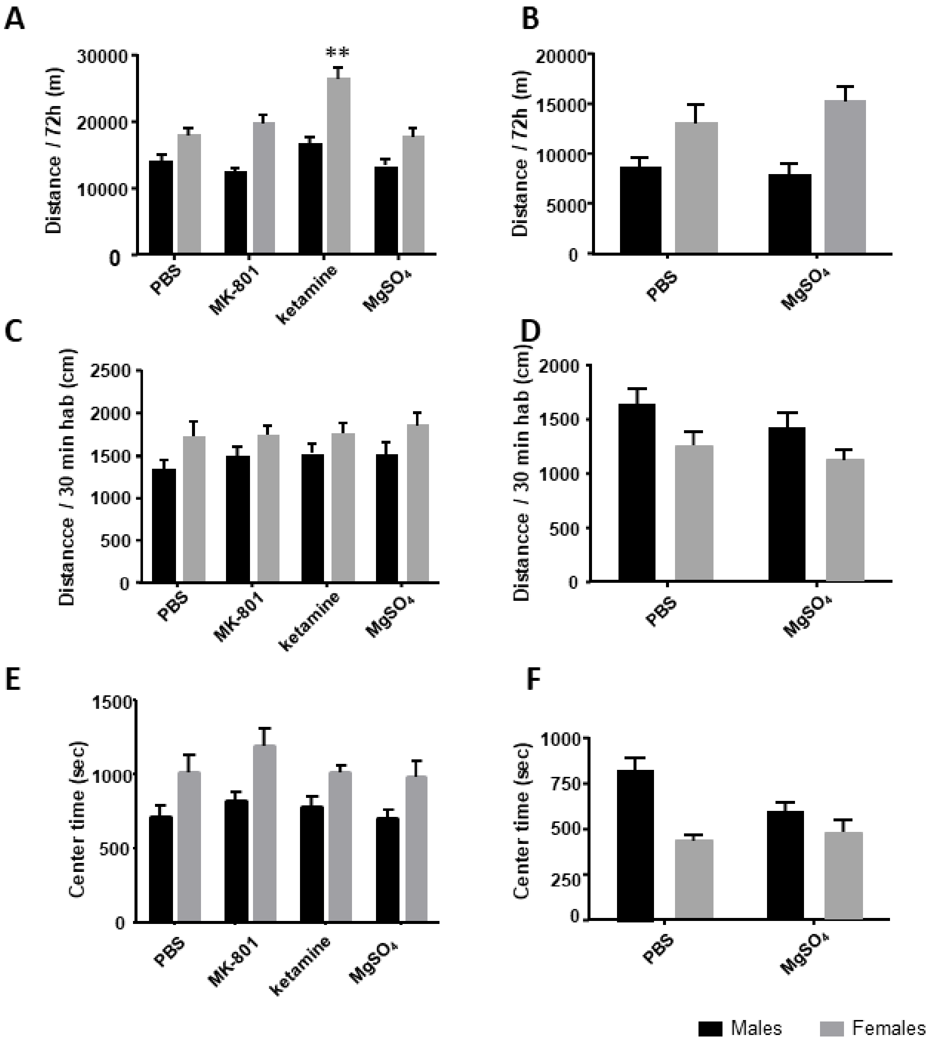

Figure S3. Effects of neonatal injection of PBS (controls), MK-801, ketamine or $MgSO_4$ in adult behavior. (**A**) Distance ran in freely access wheel for 72 hours in 8 sex and acute treatment groups. (**B**) Distance ran in freely access wheel for 72 hours in 4 sex and repeated treatment groups. (**C**) Distance ran in open field 30 min in 8 sex and acute treatment groups. (**D**) Distance ran in open field 30 min in 4 sex and repeated treatment groups. (**E**) Time spent in center area of open field device for 30 min in 8 sex and acute treatment groups. (**F**) Time spent in center area of open field device for 30 min in 4 sex and repeated treatment groups. **; $p < 0.01$ *vs* sex matched Controls according to Bonferroni post two-way ANOVA.

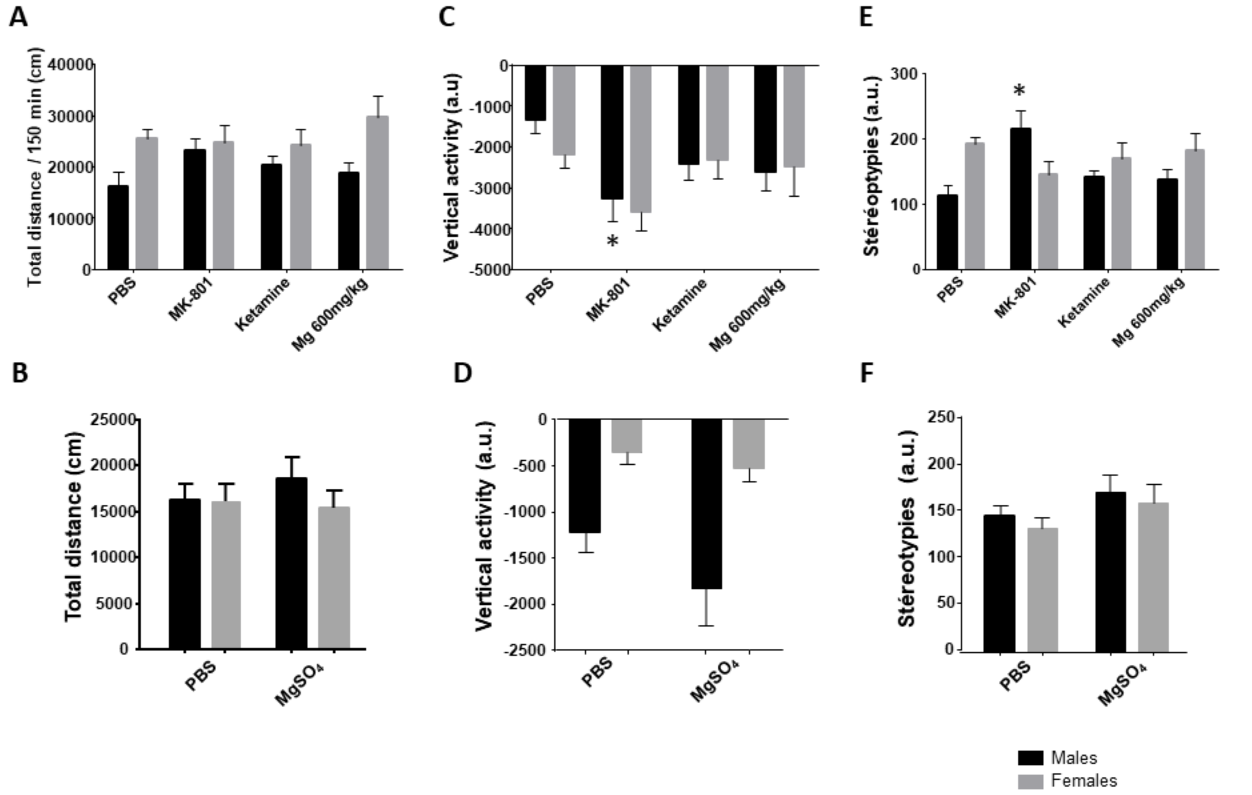

Figure S4. Effects of acute MK-801 induced locomotor activity in adult mice treated neonatally with PBS, MK-801, ketamine or $MgSO_4$ single administration at P5 or repeatedly from day 5 to day 9. (**A**) Total distance ran in the 150 min following MK-801 by adult mice treated at P5. (**B**) Total distance ran in the 150 min following MK-801 by adult mice treated repeatedly treated from P5 to P9 with PBS or $MgSO_4$. (**C**) Vertical exploration in the 150 min following MK-801 by adult mice treated at P5. (**D**) Vertical exploration in the 150 min following MK-801 by adult mice treated repeatedly. (**E**) Number of stereotypies in the 150 min following MK-801 by adult mice treated at P5. (**F)** Number of stereotypies in the 150 min following MK-801 by adult mice treated by repeatedly. *; $p < 0.05$ *vs* sex matched Controls according to Bonferroni post two-way ANOVA

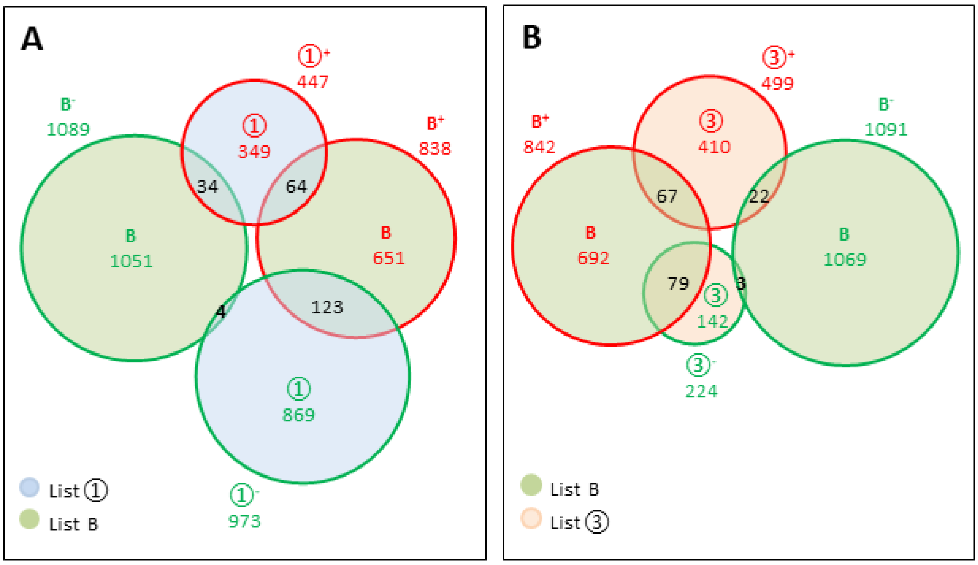

Figure S5. Comparative distribution (number of genes affected) of transcription effects of HI and/or $MgSO_4$ depending on sense of the effects. **(A)** Distribution of inductions and repressions of lists A and B. **(B)** Comparison of $MgSO_4$ interference with HI effects (List B) and in HI effects in Ctrl mice at P5 (List ③). Disk surfaces are proportional to gene numbers, intersections indicate number of genes in common. Red or green circling indicate induction and repression sub-lists, respectively. Circle fillings refer to experiment 1 (blue), 2 (green) and 3 (orange).

HI (list ③)

Early

- EGF Signaling
- MSP-RON Signaling In Cancer Cells Pathway
- HIF1Î± Signaling
- Unfolded protein response
- mTOR Signaling

Lasting

- ILK Signaling
- Hepatic Fibrosis Signaling Pathway

Late

- Role of PKR in Interferon Induction and Antiviral Response
- ERK/MAPK Signaling
- Role of IL-17F in Allergic Inflammatory Airway Diseases
- cAMP-mediated signaling
- Superpathway of Cholesterol Biosynthesis

MgHI (List B)

Early

- IGF-1 Signaling
- IL-2 Signaling
- Role of PI3K/AKT Signaling in the Pathogenesis of Influenza
- Leptin Signaling in Obesity
- Insulin Receptor Signaling
- Nitric Oxide Signaling in the Cardiovascular System
- Androgen Signaling
- RANK Signaling in Osteoclasts
- Relaxin Signaling
- Netrin Signaling
- Renal Cell Carcinoma Signaling
- CD28 Signaling in T Helper Cells
- GDNF Family Ligand-Receptor Interactions
- fMLP Signaling in Neutrophils
- GM-CSF Signaling
- Role of NFAT in Cardiac Hypertrophy
- FGF Signaling
- NGF Signaling
- Role of NFAT in Regulation of the Immune Response
- NRF2-mediated Oxidative Stress Response
- GÎ±q Signaling
- JAK/Stat Signaling
- Dendritic Cell Maturation
- Thyroid Cancer Signaling
- Endocannabinoid Developing Neuron Pathway
- Integrin Signaling
- G Beta Gamma Signaling
- PFKFB4 Signaling Pathway
- Apelin Endothelial Signaling Pathway
- Thrombin Signaling
- Sphingosine-1-phosphate Signaling
- Huntington's Disease Signaling

Lasting

- Reelin Signaling in Neurons

Both

Early

- IL-8 Signaling
- RhoGDI Signaling
- NRF2-mediated Oxidative Stress Response

Anticipated

- Neuroinflammation Signaling Pathway
- PTEN Signaling
- Melanocyte Development and Pigmentation Signaling
- Opioid Signaling Pathway
- CREB Signaling in Neurons
- P2Y Purigenic Receptor Signaling Pathway
- Dopamine-DARPP32 Feedback in cAMP Signaling
- Tec Kinase Signaling
- Ephrin Receptor Signaling
- NF-Î°B Signaling
- Synaptic Long Term Potentiation
- Neuropathic Pain Signaling In Dorsal Horn Neurons
- Renin-Angiotensin Signaling
- Synaptogenesis Signaling Pathway

Shortened

- Estrogen Receptor Signaling
- Cardiac Hypertrophy Signaling
- Phospholipase C Signaling
- PI3K Signaling in B Lymphocytes
- White Adipose Tissue Browning Pathway
- B Cell Receptor Signaling
- Cholecystokinin/Gastrin-mediated Signaling
- HMGB1 Signaling
- Senescence Pathway
- CXCR4 Signaling
- Acute Phase Response Signaling
- Corticotropin Releasing Hormone Signaling

Lasting

- Signaling by Rho Family GTPases

Figure S6. Kinetics of HI transcription effects identified by significantly enriched IPA® pathways obtained specifically in Ctrl (List ③), in MgSO4 pretreated mice (List B) or in both groups. Early effects were observed only 3h after HI, Lasting effects were observed 3h and 12h after HI; Late effects were observed only 12h after HI. Anticipated effects were observed in list B after 3h while after 12h in List ③, Shortened effects were observed in B after 3h while were lasting effects in ③.

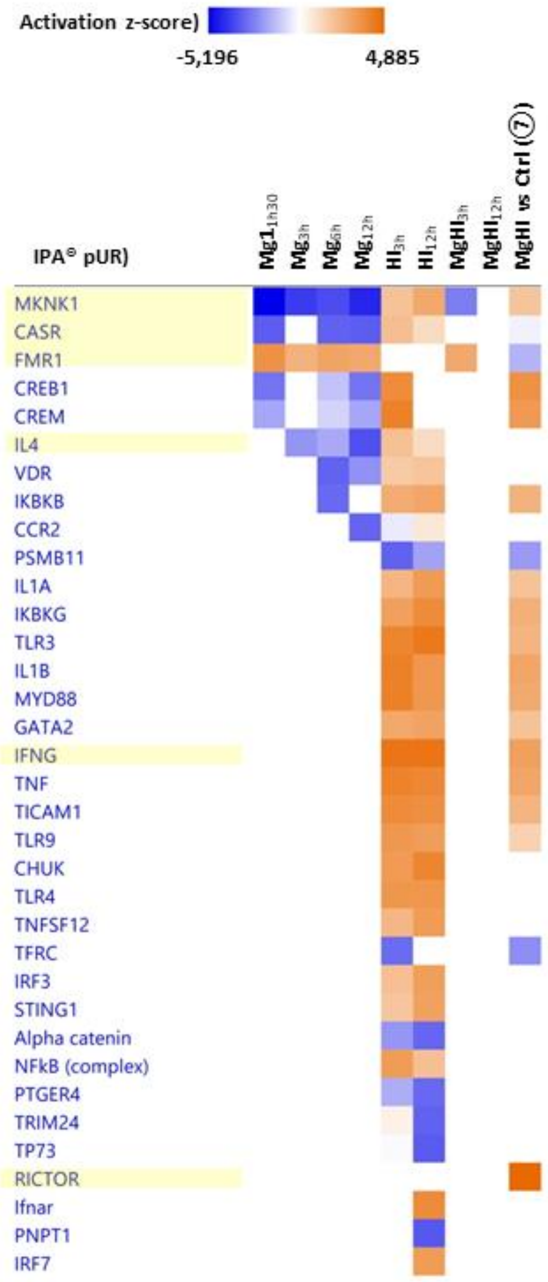

Figure S7. IPA determination of putative upstream regulators (pUR) of genes affected by $MgSO_4$ single injection. Blue color indicate inhibitions and orange indicates activations. Color intensity refer to Z-score. Filtering was set at $p < 1 \times 10^{-2}$ in Benyamini-Hochberg, and │z-score│>3 . yellow background indicates special interest genes exhibiting diverging sense of variation depending on the conditions.

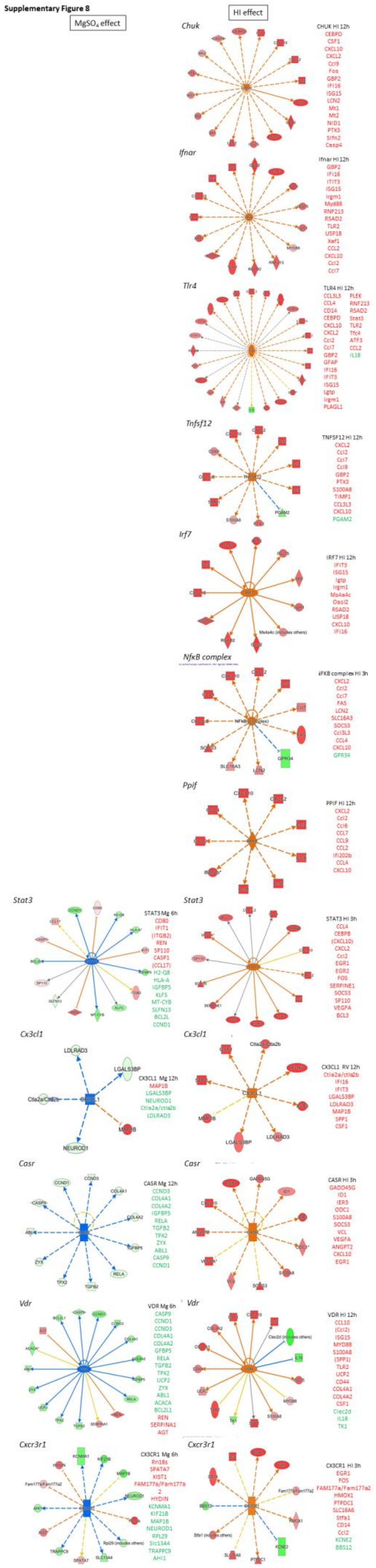

Figure S8 Upstream Regulators (pUR) putatively activated after HI on the basis of transcription effects that did not appear in MgHI group. Left column illustrates eventual detection of $MgSO_4$ effect in Ctrl mice, and right column illustrates detection oh HI effects in Ctrl mice. pUR activation in center of circles are indicated by orange background and pUR inhibition by blue background. Gene series on which uPA were identified appear in red or green background (color intensity indicating inductions or repressions, respectively). Arrows in orange indicate gene activation coherent with uPR sense of regulation. Blue arrows indicate gene repression coherent with pUR sense of regulation. Yellow arrows indicate gene observed sense of variation incoherent with pUR sense of regulation. Grey arrows indicate genes related to pUR although with no sense of variation described. Genes names indicated in red or green were induced or repressed, respectively.

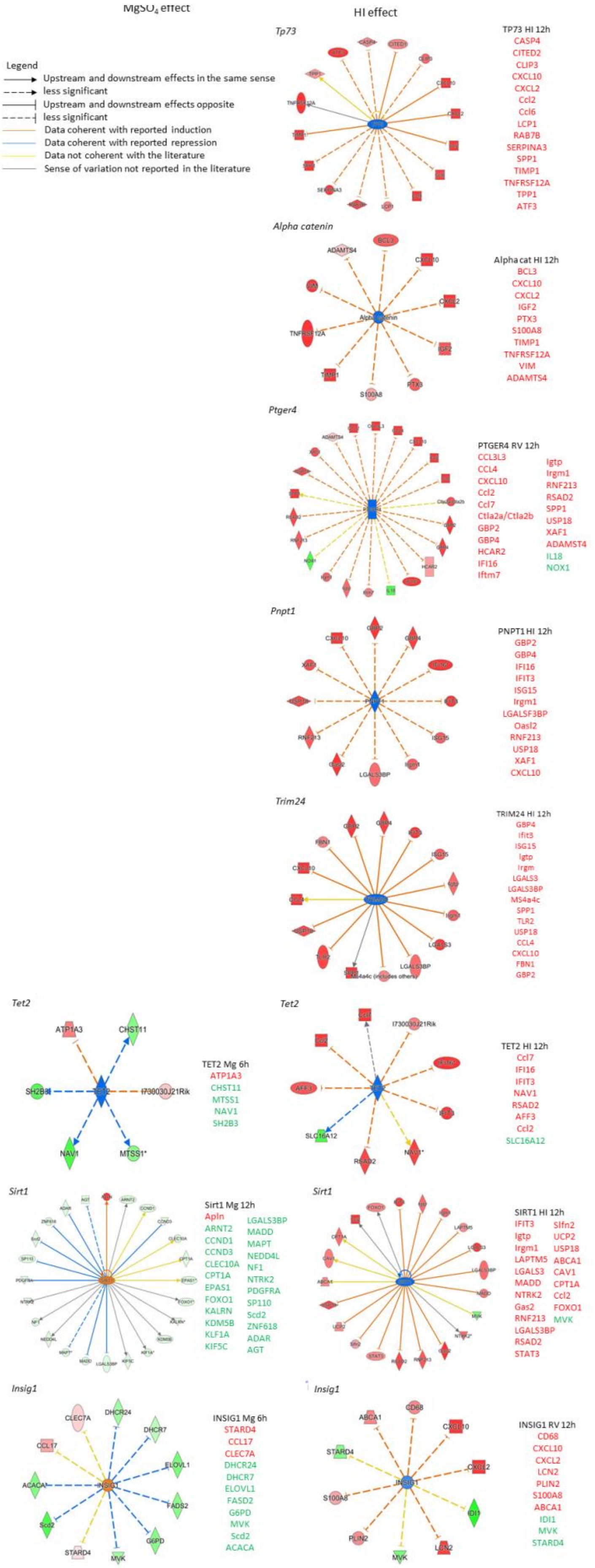

Figure S9. Upstream Regulators (pUR) putatively inhibited after HI on the basis of transcription effects that did not appear in MgHI group. Left column illustrates detection in MgSO4 effect in Ctrl mice, and right column illustrates detection oh HI effects in Ctrl mice. pUR inhibited in center of circles are indicated by blue background. For color details see text Figure S8.

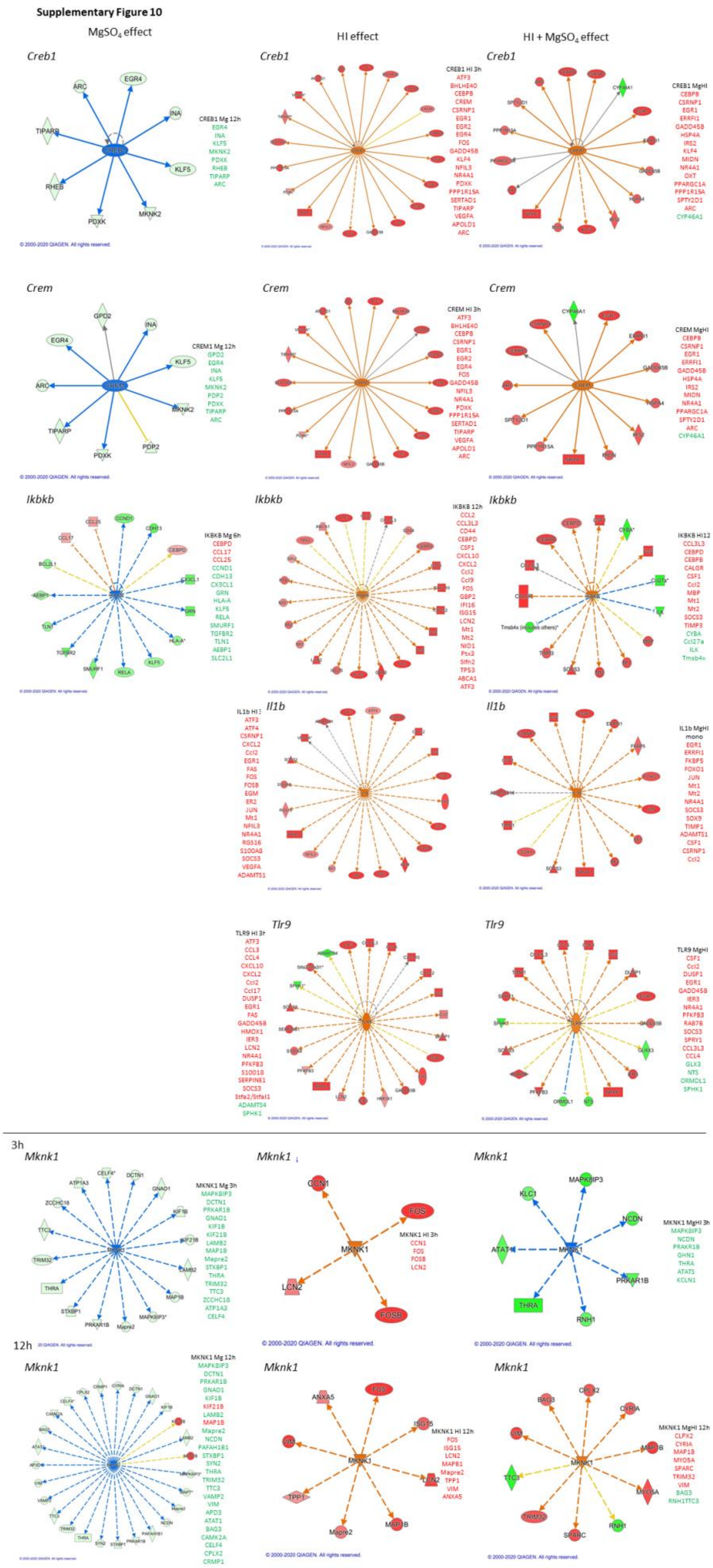

Figure S10. Upstream Regulators (pUR) putatively activated after HI that did not appear affected in MgHI group. Some appeared inhibited by $MgSO_4$ effect in Ctrl mice (left column). Center column illustrates detection of HI effects in Ctrl mice. Right column illustrates behavior in MgHI group. Particular situation of *Mknk1* is illustrated at bottom of the figure, showing early prevention by $MgSO_4$ of HI effects after 3h and independently of large effects of $MgSO_4$ alone after 12h, and its near absence of prevention of HI 12h after insult. For color details see text of Figure S8.
